# RPITER: A Hierarchical Deep Learning Framework for ncRNA–Protein Interaction Prediction

**DOI:** 10.3390/ijms20051070

**Published:** 2019-03-01

**Authors:** Cheng Peng, Siyu Han, Hui Zhang, Ying Li

**Affiliations:** College of Computer Science and Technology, Key Laboratory of Symbol Computation and Knowledge Engineering of Ministry of Education, Jilin University, Changchun 130012, China; pengcheng2114@mails.jlu.edu.cn (C.P.); hansy15@mails.jlu.edu.cn (S.H.); huizhang16@mails.jlu.edu.cn (H.Z.)

**Keywords:** ncRNA–protein interaction prediction, ncRNA, deep learning, CNN

## Abstract

Non-coding RNAs (ncRNAs) play crucial roles in multiple fundamental biological processes, such as post-transcriptional gene regulation, and are implicated in many complex human diseases. Mostly ncRNAs function by interacting with corresponding RNA-binding proteins. The research on ncRNA–protein interaction is the key to understanding the function of ncRNA. However, the biological experiment techniques for identifying RNA–protein interactions (RPIs) are currently still expensive and time-consuming. Due to the complex molecular mechanism of ncRNA–protein interaction and the lack of conservation for ncRNA, especially for long ncRNA (lncRNA), the prediction of ncRNA–protein interaction is still a challenge. Deep learning-based models have become the state-of-the-art in a range of biological sequence analysis problems due to their strong power of feature learning. In this study, we proposed a hierarchical deep learning framework RPITER to predict RNA–protein interaction. For sequence coding, we improved the conjoint triad feature (CTF) coding method by complementing more primary sequence information and adding sequence structure information. For model design, RPITER employed two basic neural network architectures of convolution neural network (CNN) and stacked auto-encoder (SAE). Comprehensive experiments were performed on five benchmark datasets from PDB and NPInter databases to analyze and compare the performances of different sequence coding methods and prediction models. We found that CNN and SAE deep learning architectures have powerful fitting abilities for the *k*-mer features of RNA and protein sequence. The improved CTF coding method showed performance gain compared with the original CTF method. Moreover, our designed RPITER performed well in predicting RNA–protein interaction (RPI) and could outperform most of the previous methods. On five widely used RPI datasets, RPI369, RPI488, RPI1807, RPI2241 and NPInter, RPITER obtained AUC of 0.821, 0.911, 0.990, 0.957 and 0.985, respectively. The proposed RPITER could be a complementary method for predicting RPI and constructing RPI network, which would help push forward the related biological research on ncRNAs and lncRNAs.

## 1. Introduction

In human genome, protein-coding genes only account for about 2% and the vast majority are non-coding RNAs (ncRNAs), which directly function at the RNA level [1,2]. Based on transcript lengths, ncRNAs could be roughly divided into small ncRNAs below 200 nucleotides, such as siRNAs, miRNAs, and piRNAs, and long non-coding RNAs (lncRNAs) over 200 nucleotides [3,4,5,6,7]. Recently, increasing evidence suggests that lncRNAs play a crucial role in the physiological processes, such as epigenetic regulation of gene expression [8], cell cycle regulation [9], and chromatin modification [10], as well as pathological processes, such as cancer [11,12,13], diabetes [14], and Alzheimer’s disease [15]. Nearly all lncRNAs function by interacting with corresponding RNA-binding proteins [16,17,18], and ncRNA–protein interactions play an important role in transcriptional and post-transcriptional gene regulation. Therefore, the effective identification of RNA–protein interaction (RPI) is essential for pushing forward our currently still limited understanding of the biological functions of ncRNA.

Since the experimental techniques for detecting RPIs are expensive and time-consuming, many computational methods have been proposed for RPI prediction in recent years. Bellucci et al. developed catRAPID [19,20] based on the physiochemical properties of protein and RNA including secondary structure, hydrogen bonding and van der Waals propensities. Muppirala et al. [21] put forward RPISeq, which feeds the sequence coding vectors of RNA and protein by conjoint triad feature (CTF) [22] to random forest (RF) and support vector machine (SVM) to make predictions. Wang et al. [23] proposed methods based on Naive Bayes (NB) and Extended NB (ENB) classifiers and performed similar work as Muppirala et al. Lu et al. [24] created a method named lncPro, which is based on Fisher linear discriminant approach and uses secondary structure, hydrogen-bond and van der Waals propensities as input features. RPI-Pred [25] combines the primary sequence information and high-order 3D structure of RNA and protein to predict RPIs based on SVM. IPMiner [26] uses the stacked auto-encoder (SAE) in deep learning to give high-level representations of the RNA and protein coding features by CTF, then predicts the RNA–protein interactions by RF classifier, and finally further improves the prediction accuracy by logistic regression (LR)-based model ensemble method.

Nonetheless, the above methods have limitations in two main respects. First, the sequence coding methods still have room for improvement. The widely-used sequence coding method of CTF only counts the 3-mer frequency of protein and 4-mer frequency of RNA to form 343-dimensional protein coding vector and 256-dimensional RNA coding vector. CTF ignores the 1–2-mer protein sequence information and 1–3-mer RNA sequence information. Meanwhile, CTF does not take sequence structure information into account. According to the consensus that structure determines function in biological field, it is important to take the sequence structure information into consideration in inferring the RNA–protein interactions. Although RPI-Pred considers the sequence structure information, it only combines seven classes of clustered amino acids with 16 kinds of protein structure blocks to form a 112-dimensional protein coding vector and combines four classes of nucleotides with five kinds of RNA structures to form a merely 20-dimensional RNA coding vector. The too short encoding vectors in RPI-Pred may fail to contain sufficient sequence information. The limited sequence information in the feature vectors would restrict the prediction performance of the subsequent classification model. Therefore, it is worth exploring more powerful sequence coding methods to effectively integrate primary sequence information and sequence structure information; Second, nearly all previous methods rely on conventional machine learning techniques to build prediction models for RNA–protein interaction. Previous studies rely on models such as RF, SVM and NB to implement interaction predictions: RPISeq employs RF and SVM; IPMiner uses RF in basic models and LR in ensemble model; and RPI-Pred employs SVM. However, deep learning provides an approach to more effectively extracting features from inputs and forming high-level representations. Moreover, deep learning-based methods have achieved state-of-the-art performance in various biological sequence analysis problems [27,28,29,30,31,32].

Recently, deep learning has obtained great success in a series of issues, such as image recognition [33,34], speech recognition [35], machine translation [36] and so on. In the field of bioinformatics, increasing works based on deep learning methods are also emerging. DeepBind [28] uses deep convolution neural network (CNN) to catch sequence motifs and overcomes the conventional methods in predicting the sequence specificities of DNA- and RNA-binding proteins. DeepSEA [29] depends on CNN to analyze sequence information and performs well in predicting the effects of noncoding variants. DeeperBind [31] adds long short-term memory (LSTM) [30] network layers on the basis of DeepBind to enhance its effectiveness by capturing the positional dynamics in sequences. TITER [37] takes advantage of CNN and LSTM to analyze the input of protein sequence vectors by one hot encoding and attains the goal of predicting the translation initiation sites. Jurtz et al. [27] introduced several protein sequence encoding methods and discussed how to use deep learning techniques including CNN, LSTM and attention mechanism to tackle the biological sequence problems such as prediction of subcellular localization and protein secondary structure. Xu et al. introduced a novel DNA sequence encoding method by training *k*-mer embedding with Glove [38], a word embedding approach in natural language processing (NLP) field, and then predicted chromatin accessibility based on the proposed encoding method and via CNN and LSTM network. In view of the state-of-the-art performance of deep learning-based methods on all kinds of biological sequence problems, it is feasible and valuable to develop the specific deep learning model to implement the RPI prediction.

Deep learning allows computational models that are composed of multiple processing layers to automatically discover representations of data with multiple levels of abstraction, which is one major advantage compared with the domain-specific feature engineering essential for conventional machine learning methods [39]. CNN in deep learning is good at extracting features from input data, and multiple convolution and pooling layers enable the CNN network to form different levels of feature abstraction. When dealing with raw biological sequence data, CNN is suited to locate motifs. Recurrent neural network (RNN) in deep learning processes every step in the sequential input recurrently, thus is ideally applied to speech, text and biological sequence data. LSTM, a variant of RNN with the memory cell, is a popular architecture due to its effectiveness in learning the long-term sequence dependency. Word2vec [40], similar to Glove, is an efficient approach in NLP for computing numeric vector representations of words, word embedding, from raw text. If we regard the *k*-mer information of biological sequence data as the word in natural language, we can naturally train the *k*-mer embedding using the word2vec tool, which could provide a promising sequence encoding method. SAE is composed of multiple auto-encoders by layer-wise unsupervised learning [41]. It can automatically learn high-level features from raw data to form reduced dimensional representation, which was used by Sun et al. [42] to process protein sequence vector coded by conjoint triad feature method and auto-covariance method to finally predict protein–protein interactions.

**Figure 1:**
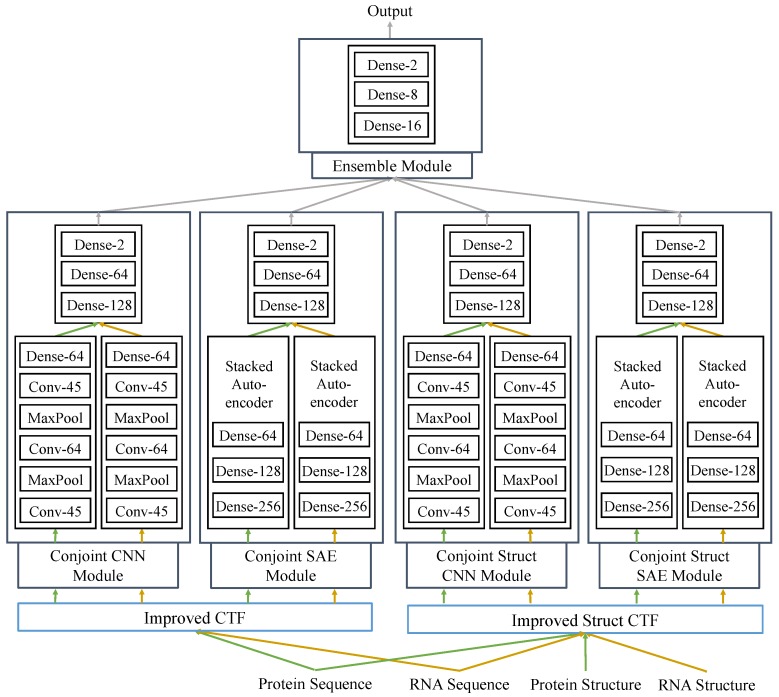
The flowchart of RPITER. The input of RPITER involves two sequence coding parts. The architecture of RPITER consists of four basic modules and an ensemble module. Dense-N means a fully-connected layer with N neurons, while Conv-M indicates a convolution layer with M filters.

In this study, we proposed a fully deep learning-based hierarchical model, RPITER, which utilizes the sequence and structure information of RNA and protein to predict the ncRNA–protein interactions. The whole proposed model consists of four modules for analyzing the inputs from two sequence coding parts and an ensemble module for integrating the outputs from basic modules to make a comprehensive prediction result. The whole architecture is shown in Figure 1. First, apply two sequence encoding methods to code the RNA and protein sequences with or without structure information; Second, use CNN and SAE-based modules to form high-level feature representations and output prediction results. Third, rely on ensemble module to combine the previous prediction results of four basic modules and further improve the prediction performance.

## 2. Results

Comprehensive experiments were performed to compare the performance of different sequence coding methods and RPI prediction methods. We employed six metrics to compare the method performance, namely accuracy (Acc), sensitivity (Sn), specificity (Sp), precision (Pre), Matthews correlation coefficient (MCC), and AUC (the area under the receiver operating characteristic curve (ROC)).

### 2.1. Performance Comparison between Different Sequence Coding Methods

**Figure 2:**
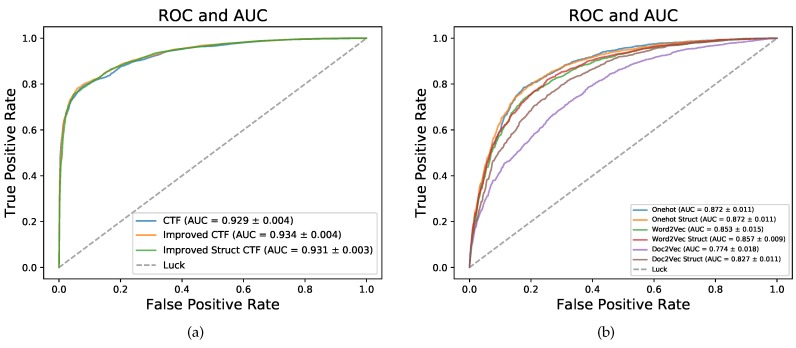
ROC and AUC comparison between different sequence coding methods on dataset RPI2241 by five-fold cross validation. (**a**) CTF, Improved CTF and Improved Struct CTF; (**b**) One hot, word2vec and doc2vec.

We improved the sequence coding method CTF by complementing more primary sequence information and sequence structure information, noted as Improved CTF and Improved Struct CTF (described in detail in Section 4.2). The performances of three sequence coding methods in five-fold cross validation (CV) on dataset RPI2241 are shown in Table 1 and Figure 2a. CTF yielded an Acc of 0.848, Sn of 0.826, Sp of 0.869, Pre of 0.864, MCC of 0.697 and AUC of 0.929. Improved CTF and Improved Struct CTF achieved the same Acc of 0.852, 0.004 higher than the 0.848 obtained by CTF. For Sn, Sp, Pre, MCC and AUC, Improved CTF and Improved Struct CTF also achieved similar performances and showed small advantages over CTF. As shown in Figure 2a, the ROC curves of three methods were very close but the curves of Improved CTF (orange) and Improved Struct CTF (green) were slightly higher than the curve of CTF (blue).

Moreover, we tried three commonly used deep learning sequence coding methods: one hot, word2vec and doc2vec [43]. One hot coding encodes each element in sequence into a binary vector and then concatenates all binary vectors successively to form the final sequence coding representation. One hot coding is extensively used for sequence coding in the deep learning-based models for biological sequence analysis [27,31,37]. Word2vec coding views *k*-mer in biological sequence as word in natural language and encodes the biological sequence by concatenating the *k*-mer representation vectors successively. Doc2vec coding directly transforms a biological sequence into a fix length vector of its embedding representation. The sequence structure information has also been considered in these three kinds of coding methods (the coding methods with “Struct” in name in Figure 2b). The ROC and AUC of these coding methods on dataset RPI2241 in five-fold CV are shown in Figure 2b. On RPI2241, one hot coding is superior to word2vec coding for having higher AUC, and word2vec coding is better than doc2vec coding. The sequence structure information notably improved the performance of doc2vec coding. The highest AUC achieved by these three coding methods is 0.872 (one hot coding), but the CTF, Improved CTF and Improved Struct CTF coding methods obtained obviously better AUC of 0.929, 0.934 and 0.931, respectively.

### 2.2. Performance Comparison between Different Basic Prediction Models

**Figure 3:**
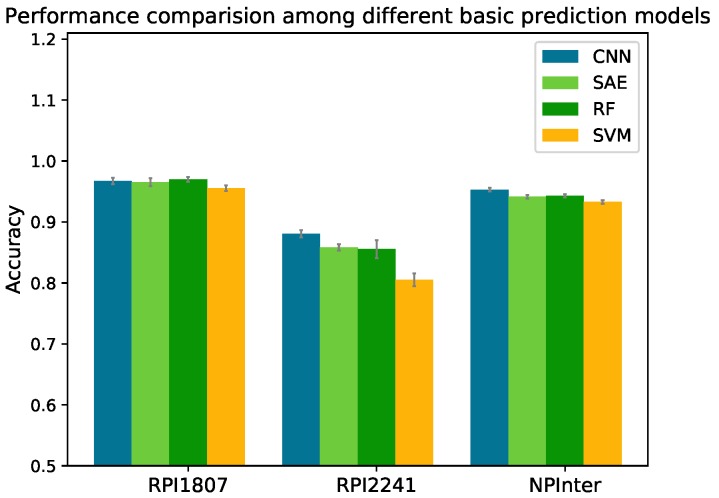
Performance comparison among different basic prediction models on datasets RPI1807, RPI2241 and NPInter by five-fold cross validation.

RF and SVM were employed to build RPISeq’s classifiers; IPMiner fed the RNA and protein features into RF to make predictions. Our proposed RPITER employed CNN and SAE architectures in four basic prediction modules to map input features into high-level feature representations and generate prediction results. RPISeq, IPMiner and RPITER all rely on the CTF form sequence coding features. Therefore, we compared the performances of different prediction models, namely RF, SVM, CNN and SAE, using same CTF coding features on datasets RPI1807, RPI2241 and NPInter. For SVM, we adopted the parameter setting in [21] (kernel = “polynomial”, degree = 2, C = 1.0, and tolerance = 0.001). For RF, we set the number of decision trees to 100. The Acc of four basic prediction models on the three datasets in five-fold CV are shown in Figure 3. The detailed performances of these methods on dataset NPInter are listed in Table 2.

On dataset NPInter, CNN obtained an Acc of 0.953 with 0.012, 0.010 and 0.020 increase over SAE (0.941), RF (0.943) and SVM (0.933), respectively. On dataset RPI1807, the differences of accuracy among CNN (0.967), SAE (0.965) and RF (0.970) were slight, and SVM (0.956) obtained the lowest accuracy. On dataset RPI2241, SAE, RF and SVM achieved Acc of 0.859, 0.855 and 0.805, respectively, whereas CNN model yielded an Acc of 0.887 (about 0.03 increase over SAE and RF, and 0.08 increase over SVM).

### 2.3. Performance Comparison between Different Modules of RPITER

**Figure 4:**
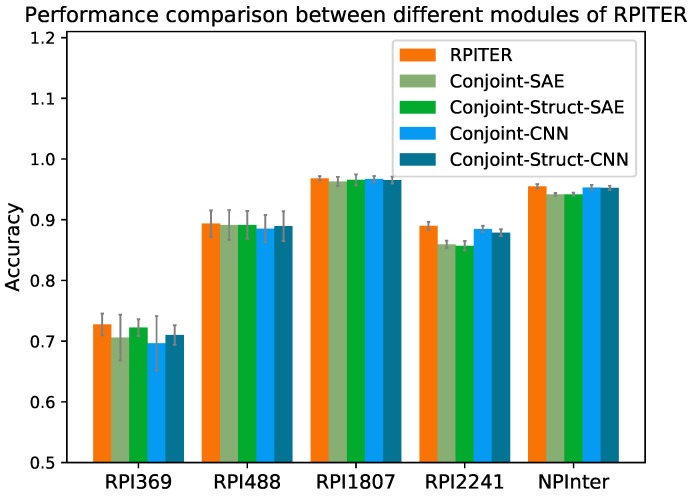
Performance comparison among different prediction modules of RPITER.

Our whole model RPITER consist of four basic prediction modules, namely Conjoint-CNN, Conjoint-SAE, Conjoint-Struct-CNN, and Conjoint-Struct-SAE (described in detail in Section 4.4). The prediction accuracy of our four basic modules and whole architecture RPITER on five benchmark datasets by five-fold CV are shown in Figure 4.

Comparing the performance of modules using different architectures of CNN and SAE but the same sequence coding inputs, it was found that the CNN-based modules have advantages over the SAE-based modules when the training samples are sufficient. On the two smallest datasets RPI369 and RPI488, the Acc of Conjoint-CNN and Conjoint-Struct-CNN were slightly inferior to Conjoint-SAE and Conjoint-Struct-SAE. On the two medium size datasets RPI1807 and RPI2241 and the largest dataset NPInter, the Acc of Conjoint-CNN and Conjoint-Struct-CNN had advantages over Conjoint-SAE and Conjoint-Struct-SAE. On RPI2241, Conjoint-CNN and Conjoint-Struct-CNN obtained 0.885 and 0.879 Acc, respectively, which notably better than Conjoint-SAE (0.859) and Conjoint-Struct-SAE (0.857). On NPInter, Conjoint-CNN (0.953) and Conjoint-Struct-CNN (0.953) both yielded a 0.011 higher Acc than Conjoint-SAE (0.942) and Conjoint-Struct-SAE (0.942).

No individual module can always surpass all other modules on all datasets. The architecture of our proposed RPITER combines the advantages of four basic modules with different architectures and sequence coding methods, which can provide a more comprehensive RPI prediction result.

### 2.4. Performance Comparison with Other Previous RNA–Protein Interaction (RPI) Prediction Methods

**Figure 5:**
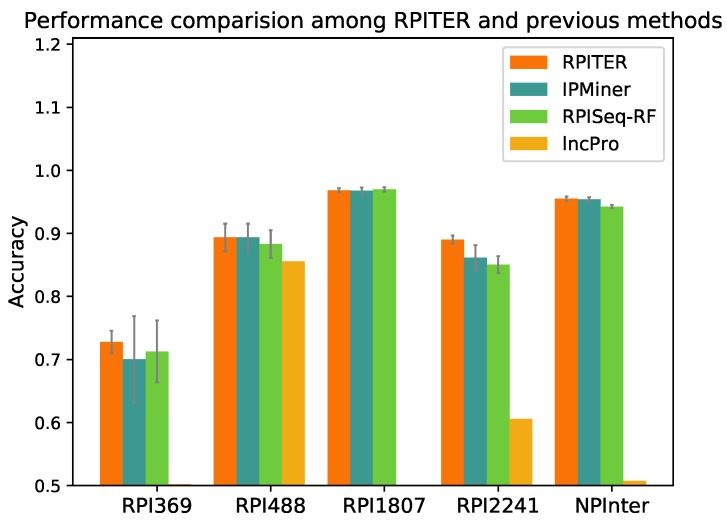
Performance comparison among different RNA–protein interaction (RPI) prediction methods on datasets RPI369, RPI488, RPI1807, RPI2241 and NPInter.

To further evaluate our proposed RPITER, we also compared our method with other previous RPI prediction methods. Since catRAPID and RPI-Pred do not offer standalone packages, we could only compared our methods with RPISeq, lncPro and IPMiner. For RPISeq, Muppirala et al. [21] proposed RPISeq-RF and RPISeq-SVM using RF and SVM, respectively, but RPISeq-RF outperformed RPISeq-SVM on RPI369 and RPI2241 according to their reported performances. Thus, in this study, RPISeq-RF was chosen for comparison. lncPro only provided the prediction source code of a trained model on their dataset. Thus, we directly tested the performance of their model on our five benchmark datasets. For IPMiner, RPISeq-RF and RPITER, the same data pre-processing and *k*-mer counting manner were conducted under the same five-fold CV condition. The prediction accuracy of the above four methods on five datasets are shown in Figure 5, and the detailed performance are listed in Table 3.

As shown in Figure 5, on datasets RPI369 and RPI2241, RPITER had a notable accuracy increase over all other methods, and the standard deviations of accuracy in five-fold CV (gray lines on the top of accuracy columns) were obviously smaller than IPMiner and RPISeq-RF. On datasets RPI488 and NPInter, RPITER also performed better than RPISeq-RF and lncPro and had similar prediction accuracy as IPMiner. On dataset RPI1807, RPITER, IPMiner and RPISeq-RF achieved similar accuracy and all outperformed lncPro. Moreover, on datasets RPI369, RPI1807, RPI2241 and NPInter, RPITER yielded the highest Sn and AUC among all methods. It should be noted that the dataset used to train the lncPro model overlapped with RPI488 [26]. Thus, lncPro performed well on RPI488 but showed poor performances on other datasets. IPMiner employed complex stacked ensembling [26] technique to increase the performance over its basic predictors including RPISeq-RF. In implementation, each of the three basic predictors of IPMiner was trained three times by a three-fold cross validation to generate the training and testing data for the LR-based ensemble part. As shown in Figure 5, this complicated ensemble manner brought about noteworthy Acc increase over RPISeq-RF on datasets RPI488 (0.010), RPI2241 (0.010) and NPInter (0.014). Nonetheless, the ensemble manner was too time-consuming for deep learning-based models. In contrast, RPITER directly trained each basic module one time, and then concatenated the outputs of four basic modules and trained the whole architecture one time again to make RPI predictions.

## 3. Discussion

In this study, we proposed a hierarchical deep learning-based framework RPITER, which contains two sequence coding parts as inputs and involves two basic CNN and SAE network architectures to generate comprehensive prediction result.

For sequence coding, we adjusted the CTF coding methods by adding more primary sequence information and sequence structure information into the coding vectors. According to the performance comparison on dataset RPI2241, our Improved CTF and Improved Struct CTF showed advantages over the previous CTF coding method. Besides, we tried three commonly used deep learning sequence coding methods of one hot, word2vec and doc2vec, but they performed worse than CTF form coding methods in this RPI prediction problem and were abandoned in this study. The sequence structure used in our coding method was predicted by the software, thus might only contain limited information. We noticed that RPI-Pred achieved 93% Acc using the experimentally validated structure but only 83% Acc using the predicted sequence structure on dataset RPI1807 [25]. Thus, more experimentally validated sequence structure data would contribute to more effective RNA–protein interaction prediction.

For model design, we found CNN architecture has a powerful fitting ability for the *k*-mer features of protein and RNA sequence compared with SAE architecture and machine learning models RF and SVM. We infer that our CNN-based modules using convolution neural networks are more complicated than our SAE-based modules using simple fully-connected layers, thus the CNN-based modules have larger sample demands for effective training than the SAE-based modules. Nonetheless, when sufficient samples are available, our CNN-based architecture can perform better in feature extraction and high-level feature representation than the SAE-based architecture. In 2017, Sun et al. [42] employed SAE architecture to process the conjoint triad features of protein sequence for protein–protein interaction prediction. Their model would probably have a performance increase if the SAE architecture were replaced by the CNN architecture, since CNN is more effective.

The designed deep learning model RPITER could integrate the advantages of four different basic predict modules and provide a comprehensive RPI prediction result. Based on experiments on five benchmark datasets, the proposed RPITER showed good performance in predicting RPI compared with the previous methods.

## 4. Materials and Methods

### 4.1. Benchmark Datasets

We employed five benchmark datasets (Table 4) from previous studies to validate the proposed method. Datasets RPI369, RPI488, RPI1807 and RPI2241 are extracted from the RNA–protein interaction complexes in the RNA–protein interaction database PRIDB [44] or PDB [45]. These four datasets are constructed following the criterion of least atom distance: if there exist a protein atom and a RNA atom with the distance between being less than the specified distance threshold, then the protein and RNA become an interaction pair. Additionally, RPI488 is comprised of lncRNA–protein interaction pairs, that is, the RNAs are ncRNAs longer than 200 nt. In contrast, rather than judging interaction by the atom distances in RPI complexes, the dataset NPInter contains the experimentally verified interactions between ncRNAs, especially lncRNAs, and proteins from the NPInter2.0 database [46]. Because RPI369, RPI2241 and NPInter datasets lack non-interaction pairs to work as negative samples in training model, the same number of non-interaction pairs were generated by randomly pairing the RNAs and proteins in positive samples and further discarding similar known interaction pairs [21,26] (a randomly generated pair R1–P1 was discarded if there existed an interaction pair R2–P2 with R1 and R2 shared ⩾80% sequence identity and P1 and P2 shared ⩾40% sequence identity). The cd-hit and cd-hit-est programs in CD-HIT version 4.6.8 [47,48] were applied to cluster RNA and protein sequences with identity thresholds being 0.4 and 0.8, the smallest clustering cutoff values in CD-HIT tool for protein and RNA, respectively.

In this study, the protein and RNA sequence data of RPI488 and NPInter were downloaded from https://github.com/xypan1232/IPMiner. The sequence data of RPI369, RPI1807 and RPI2241 were retrieved from PDB. The structure information of protein sequence used in our study was predicted by SOPMA [49]. We employed their web server (https://npsa-prabi.ibcp.fr/cgi-bin/npsa_automat.pl?page=npsa_sopma.html) to calculate the classical three state protein secondary structures: α-helix, β-sheet and coil. For RNA structure prediction, we used RNAfold program in ViennaRNA Package version 2.4.3 [50] to calculate the two state dot-bracket representation of RNA secondary structure with min free energy. The sequence data and predicted sequence structure data of five RPI datasets are organized and available at https://github.com/Pengeace/RPITER.

### 4.2. Sequence Coding Method

To input RNA and protein sequences into deep learning or conventional machine learning models, the sequence data must first be transformed into numerical representations. Because the RNA and protein sequence length vary in a large range (0–4000) in our datasets, the commonly used fixed length sequence encoding methods for deep learning models, such as one hot encoding, do not fit our problem. Therefore, we adopted and improved the CTF method, which counts *k*-mer frequency in sequence to form fixed length vector representation.

In previous CTF methods [21,26], the 3-mer frequency of protein and 4-mer frequency of RNA are calculated to form the sequence coding vectors. For protein, 20 amino acids are classified into seven groups based on their dipole moments and the volume of side chains: {A, G, V}, {I, L, F, P}, {Y, M, T, S}, {H, N, Q, W}, {R, K}, {D, E}, and {C}. Then, each protein sequence is represented by the reduced seven-letter alphabet. Thus, by calculating the 3-mer frequency, the protein sequence can be transformed into a numeric vector with 343 ($7^{3}$) elements. For RNA, using four kinds of ribonucleotides (A, U, C, G), a RNA sequence can be represented by a numeric vector with 256 ($4^{4}$) elements.

In this study, when only considering the sequence information, we extended the range of *k* to 1–3 in the *k*-mer frequency coding process for protein, and extend the range of *k* to 1–4 in the *k*-mer frequency coding process for RNA. That is, for protein, we computed the 1-mer, 2-mer and 3-mer frequency information to form an extended coding vector with 399 ($\sum_{i=1}^{3}7^{i}$) elements. For RNA, we compute the 1-mer, 2-mer, 3-mer and 4-mer frequency information to form an extended coding vector with 340 ($\sum_{i=1}^{4}4^{i}$) elements. By adding more sequence information into its coding vector, we hoped to enhance the model input to finally improve the model prediction performance. This sequence coding method is referred as Improved CTF.

When taking the sequence structure information into consideration, we calculated the 1–3-mer frequency of protein secondary structure and the 1–4-mer frequency of RNA secondary structure to complement the sequence coding vectors. For protein, combining the 1–3-mer frequency of three kinds of secondary structure (α-helix, β-sheet and coil) with previous reduced seven-letter alphabet would generate the protein coding vector with 438 ($\sum_{i=1}^{3}3^{i}+7^{i}$) elements. For RNA, integrating the 1–4-mer frequency of two kinds of secondary structure (dot and bracket) with four ribonucleotides would produce the RNA coding vector with 370 ($\sum_{i=1}^{4}2^{i}+4^{i}$) elements. This sequence coding method is noted as Improved Struct CTF. Table 5 summaries the coding length of protein and RNA by the above three different sequence coding methods.

### 4.3. Performance Evaluation

In this study, we evaluated the performance of RPITER and other methods by six metrics, Acc, Sn, Sp, Pre, MCC, and AUC. The formulas of the first five measurements are as follows:

$$\begin{array}{cc} Acc= & \frac{TP+TN}{P+N},\;Sn=\frac{TP}{TP+FN},\\ Sp= & \frac{TN}{TN+FP},\;Pre=\frac{TP}{TP+FP},\\ MCC= & \frac{TP\cdot TN-FP\cdot FN}{\sqrt{\left(TP+FP)(TP+FN)(TN+FP)(TN+FN\right)}}, \end{array}$$

where TP and TN mean the number of correctly predicted positive and negative samples, respectively; FP and FN denote the number of wrongly predicted positive and negative samples, respectively; and *P* and *N* represent the total number of positive and negative samples, respectively. All these evaluation metrics were calculated by five-fold CV and no overlap between training and testing data.

### 4.4. Model Design

We designed a deep-learning framework, RPITER (Figure 1), to tackle the RNA–protein interaction problem. Our two sequence coding methods are used in the two sequence coding parts: Improved CTF coding and Improved Struct CTF coding. After each coding part, two different network architectures, CNN and SAE, are employed in two modules to extract features from the input and form high-level representations. In each module, the protein and RNA coding vectors are analyzed by two similar parts separately to form respective sequence embedding representations. Finally, an ensemble module integrates the outputs from the four basic modules (Conjoint-CNN, Conjoint-SAE, Conjoint-Struct-CNN, and Conjoint-Struct-SAE) to form the whole architecture of RPITER. The source code of RPITER can be freely download from https://github.com/Pengeace/RPITER.

In module Conjoint-CNN and Conjoint-Struct-CNN, first, two similar sequence embedding parts using CNN analyze the RNA and protein input vectors separately and form two sequence embeddings. Then, a three-layer fully-connected part concatenates the two sequence embeddings as input and make the interaction prediction. Each sequence embedding part with CNN has three convolution layers with filter numbers being 45, 64 and 45, thus is denoted as Conv-45-64-45. Between two convolution layers, max-pooling layer is used to reduce the representation dimension and introduce an invariance to noises. After the last convolution layer in Conv-45-64-45, the output two-dimensional tensor is flattened and further serves as the input of a fully connection layer with 128 neurons, denoted as Dense-128. Then, the two sequence embedding representations of RNA and protein are output by the two Dense-128 layers separately. Finally, a three-layer fully-connected part with 128, 64 and 2 neurons in each layer, Dense-128-64-2, takes the previous two sequence embeddings and makes the interaction prediction. In Dense-128-64-2, the input of the first Dense layer is the concatenation of the protein and RNA embeddings, and the output of the last Dense layer is the prediction result, which is further integrated by the later ensemble module.

In module Conjoint-SAE and Conjoint-Struct-SAE, first, two similar sequence embedding parts using SAE analyze the RNA and protein input vectors separately and generate two sequence embeddings. Then, a three-layer fully-connected part concatenates the two sequence embeddings as input and makes the interaction prediction. Each sequence embedding part with SAE has three fully-connected layers with neuron numbers being 256, 128 and 64, thus is denoted as Dense-256-128-64. After dimension reduction and high-level feature abstraction by the two three-layer SAE parts, the sequence embedding representations of RNA and protein are output by the last layers of two Dense-384-256-128 parts. Finally, a three-layer fully-connected part Dense-128-64-2 concatenates the previous two sequence embeddings as input for its first layer and makes the interaction prediction for a specific RNA–protein pair at the third layer. Similar to previous CNN-based modules, the prediction results of the two SAE-based modules are further integrated by the later ensemble module.

The last ensemble module concatenates the prediction results of former two CNN modules and two SAE modules as its input tensor and generates a more comprehensive prediction result for a given RNA–protein pair. The ensemble module is designed as a three-layer architecture, Dense-16-8-2, with three fully-connected layers in which the neuron numbers are 16, 8, and 2, respectively.

The four basic modules and ensemble module use the Softmax [51] activation function at their last layers to make binary predictions, and use back-propagation algorithm [52] to minimize loss function of binary cross entropy. Two optimization methods, Adam [53] and stochastic gradient descent (SGD) [54], are employed successively to train each module, among which Adam first gives the module a quick converge and then SGD is used to fine tune the module after. In Conv-45-64-45, Dense-128-64-2, and Dense-16-8-2, batch normalization [55] is employed to reduce internal covariate shift and help train the designed deep network, and ReLU [56] activation function is used to speed up the supervised train process. During the unsupervised pre-training process of the three-layer SAE, its parameters are optimized by greedy layer-wise training. To avoid over-fitting problem, the techniques of dropout [57] and early stopping [58] are also used. After each training epoch on the training data, the Acc of a training module is recorded. If the prediction Acc this time is higher than the highest Acc record in previous epochs, then the whole module parameters are saved and the highest Acc record is updated. If the highest Acc record on training data has not been updated for specific epochs, the train process is stopped and the module loads the previous lastly saved weights and tests once on the test data. Our model was implemented by the Keras2.0 library (https://keras.io/).

## Figures and Tables

**Table 1:** Performance comparison between Conjoint Triad Feature (CTF) and our two improved coding methods on dataset RPI2241 by five-fold cross validation.

Dataset	Coding Method	Acc	Sn	Sp	Pre	MCC	AUC
RPI2241	CTF	0.848	0.826	0.869	0.864	0.697	0.929
	Improved CTF	0.852	0.833	0.872	0.867	0.705	0.934
	Improved Struct CTF	0.852	0.834	0.870	0.865	0.704	0.931

**Table 2:** Performance comparison between convolution neural network (CNN), stacked auto-encoder (SAE), random forest (RF), and support vector machine (SVM) on dataset NPInter by five-fold cross validation.

Dataset	Models	Acc	Sn	Sp	Pre	MCC	AUC
NPInter	CNN	0.953	0.974	0.932	0.935	0.907	0.984
	SAE	0.941	0.967	0.915	0.920	0.884	0.982
	RF	0.943	0.945	0.941	0.941	0.886	0.943
	SVM	0.933	0.940	0.925	0.926	0.866	0.933

**Table 3:** Performance comparison of different RNA–protein interaction (RPI) prediction methods on five benchmark datasets by five-fold cross validation.

Dataset	Method	Acc	Sn	Sp	Pre	MCC	AUC
RPI369	RPITER	**0.728**	**0.797**	0.659	0.701	**0.461**	**0.821**
	IPMiner	0.700	0.784	0.560	0.840	0.428	0.700
	RPISeq-RF	0.713	0.716	**0.702**	**0.724**	0.426	0.713
	lncPro	0.502	0.237	0.771	0.512	0.009	0.468
RPI488	RPITER	**0.893**	0.839	**0.947**	0.943	**0.793**	0.911
	IPMiner	**0.893**	**0.946**	0.835	**0.951**	**0.793**	0.893
	RPISeq-RF	0.883	0.928	0.831	0.935	0.771	0.883
	lncPro	0.856	0.770	**0.947**	0.940	0.725	**0.929**
RPI1807	RPITER	0.968	**0.986**	0.946	0.959	0.936	**0.990**
	IPMiner	0.968	0.965	**0.978**	0.955	0.935	0.966
	RPISeq-RF	**0.970**	0.970	0.976	**0.962**	**0.939**	0.969
	lncPro	0.472	0.445	0.506	0.532	-0.049	0.506
RPI2241	RPITER	**0.890**	**0.917**	**0.863**	0.871	**0.781**	**0.957**
	IPMiner	0.861	0.877	0.841	**0.882**	0.724	0.861
	RPISeq-RF	0.851	0.861	0.838	0.863	0.702	0.851
	lncPro	0.606	0.518	0.695	0.632	0.216	0.644
NPInter	RPITER	0.955	**0.973**	0.937	0.939	0.910	**0.985**
	IPMiner	**0.957**	0.956	**0.958**	**0.956**	**0.914**	0.957
	RPISeq-RF	0.943	0.937	0.949	0.936	0.885	0.943
	lncPro	0.508	0.739	0.276	0.505	0.017	0.517

The boldface indicates the highest metric performance among the compared methods on specific dataset.

**Table 4:** The five benchmark RPI datasets used in this study.

Dataset	Interaction Pairs	Non-Interaction Pairs	RNAs	Proteins	Reference
RPI369	369	0	332	338	[21]
RPI488	243	245	25	247	[26]
RPI1807	1807	1436	1078	3131	[25]
RPI2241	2241	0	841	2042	[21]
NPInter	10,412	0	4636	449	[46]

RPI369, RPI2241 and NPInter lack non-interaction pairs to serve as negative training samples, thus we randomly paired the RNAs and proteins in positive interaction samples and discarded existing pairs to generate the same number of negative samples and construct the balanced training datasets [21,26].

**Table 5:** The sequence coding lengths for protein and RNA of different encoding methods.

Sequence	CTF	Improved CTF	Improved Struct CTF
RNA	$4^{4}=256$	$\sum_{i=1}^{4}4^{i}=340$	$\sum_{i=1}^{4}\left(2^{i}+4^{i}\right)=370$
Protein	$7^{3}=343$	$\sum_{i=1}^{3}7^{i}=399$	$\sum_{i=1}^{3}\left(3^{i}+7^{i}\right)=438$
